# Time-Efficient Black Blood RCA Wall Imaging at 3T Using Improved Motion Sensitized Driven Equilibrium (iMSDE): Feasibility and Reproducibility

**DOI:** 10.1371/journal.pone.0026567

**Published:** 2011-10-20

**Authors:** Jinnan Wang, Suzanne C. Gerretsen, Jeffrey H. Maki, Caroline Jaarsma, M. Eline Kooi, Daniel Herzka, Baocheng Chu, Vasily L.Yarnykh, Chun Yuan, Tim Leiner

**Affiliations:** 1 Department of Radiology, University of Washington, Seattle, Washington, United States of America; 2 Clinical Sites Research Program, Philips Research North America, Briarcliff Manor, New York, United States of America; 3 Department of Radiology and Cardiology, Cardiovascular Research Institute Maastricht (CARIM), Maastricht University Medical Center, Maastricht, The Netherlands; 4 Department of Biomedical Engineering, Johns Hopkins University School of Medicine, Baltimore, Maryland, United States of America; 5 Utrecht University Medical Center, Utrecht, The Netherlands; Virginia Commonwealth University, United States of America

## Abstract

**Background:**

The aim of this study was to explore the feasibility and reproducibility of a time-efficient coronary vessel wall measurement approach using an improved motion-sensitized driven equilibrium (iMSDE) pulse sequence.

**Methodology:**

In this study, the iMSDE pulse sequence was first optimized and then applied on a group of healthy volunteers (N = 10) to evaluate its feasibility of vessel wall visualization. The same technique was also applied on a separate group of volunteers (N = 19) for a reproducibility study by scanning the same subject in two separate sessions. The iMSDE sequence was found to provide good coronary vessel wall delineation. It was also found to provide reproducible coronary vessel wall diameter and thickness measurements in both proximal and middle segments of the right coronary artery.

**Conclusion:**

The feasibility and reproducibility of iMSDE based coronary vessel wall imaging were demonstrated for the first time, paving the way for further testing in a clinical environment for fast and accurate coronary artery disease detection.

## Introduction

Coronary artery disease (CAD) remains the number one cause of death in the Western world [1] and over half of all patients with sudden cardiac death do not experience typical symptoms such as chest pain prior to the event [2], [3]. In order to identify patients with relevant but unrecognized CAD, clinical algorithms such as the Framingham Risk Index [4] and Prospective Cardiovascular Münster Heart Study (PROCAM) [5] have been developed. Although the use of these and other risk scores is a major advance over clinical risk predictions based on relative risk estimates, they still remain imperfect for predicting cardiovascular events in individual patients [6].

To overcome this limitation, a lot of effort has been made to detect the presence of subclinical atherosclerotic lesions using imaging approaches, such as computed tomography angiography [7] and intravascular ultrasonography [8]. Magnetic resonance imaging (MRI) is an attractive alternate for this purpose as it is totally non-invasive, does not require the injection of contrast medium, and is well suited for repetitive imaging in cases where follow-up is desired. Furthermore, with excellent soft tissue contrast, MRI is capable of detecting not only luminal narrowing [9], but also positive remodeling [10] of the coronary vessel wall (CVW) [11]. The latter phenomenon has been traditionally regarded as a pivotal sign of clinically relevant future coronary artery disease [12], and might influence individual treatment decisions.

Black-blood MRI techniques, which can suppress hyperintense blood signal for better visualization of the vessel wall, are important for coronary artery vessel wall measurements. The currently used method for MR CVW imaging is based on a double inversion recovery (DIR) prepulse [11]. This technique, however, is limited in a few aspects when applied for CVW imaging. First, it requires a relatively long inversion time (TI), usually 400–500 ms, to achieve sufficient blood nulling. As a result, imaging of a single coronary artery with this method may take up to 10–15 minutes, since a repetition time (TR) of two heartbeats is required to achieve sufficient blood suppression. Second, due to the long TI time, the optimal TI time usually conflicts with the optimal trigger delay, forcing the adoption of a less optimal imaging protocol. Third, DIR based techniques can achieve only suboptimal blood suppression if the imaging plane is not perpendicular to the flow direction. As a result, when an artery with tortuous segments is imaged, flow artifacts can be observed. Furthermore, as has been shown in carotid artery imaging, the DIR technique is known to suffer from high signal-intensity slow flow artifacts at the boundary between the vessel wall and lumen, as it requires complete blood replenishing to achieve luminal blood suppression [13].

An improved motion-sensitized driven equilibrium (iMSDE) technique that was originally developed for carotid artery imaging [13], [14] has been recently shown to achieve more time efficient acquisition and more accurate depiction of the carotid artery luminal boundaries. We hypothesize that robust and reproducible in vivo MR imaging of the human CVW can be achieved in a more time-efficient manner by using the iMSDE technique. The objective of the current study was to investigate whether the iMSDE preparation [14] could also be applied for CVW imaging and whether these measurements are reproducible.

## Methods

### Study design

In the first part of the study, the iMSDE sequence was tested for feasibility and optimized to acquire CVW in a time efficient manner. After imaging parameters were determined, the reproducibility of the new method was investigated in the second part of the study. All scans were performed on a clinical 3.0T system (Philips Achieva, Release 2.5, Best, The Netherlands), using a commercially available 6-element phased-array cardiac coil. Subjects were examined in the supine position. The study was approved by the Maastricht University Medical Center review board and written informed consent of all participants was obtained prior to inclusion.

### MR Pulse Sequence

**Figure 1:**
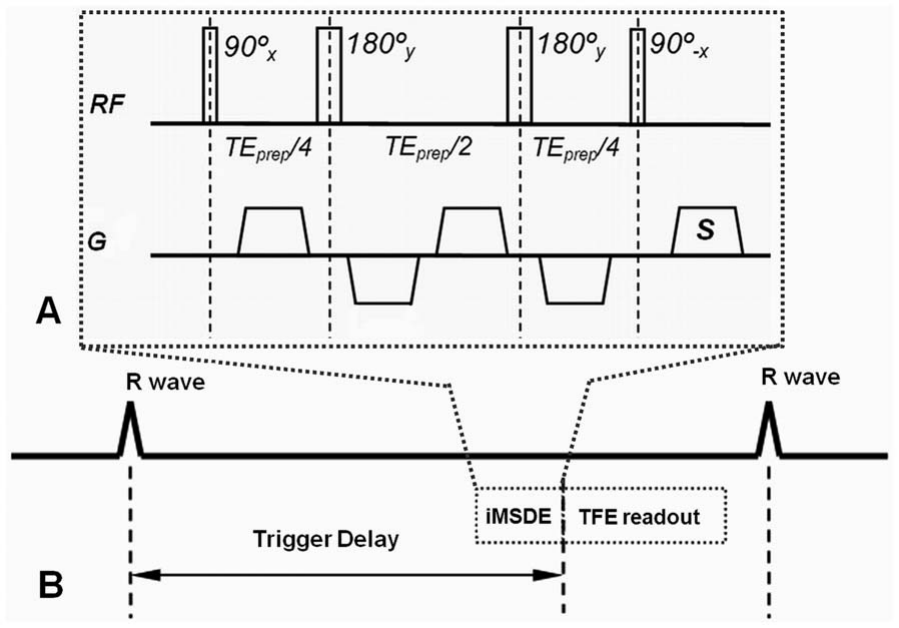
Imaging diagram of the pulse sequence used for this study. A) the improved MSDE (iMSDE) pulse sequence used to suppress blood signal; and B) the timing of iMSDE prepulse and TFE acquisition sequence in a CVW imaging process.

The pulse sequence of the iMSDE sequence is shown in Fig. 1a. Compared to the traditional MSDE [13], the iMSDE technique was selected for this study because the MLEV-4 pulse design provides better immunity to local magnetic field inhomogeneities and the new gradient arrangement is more immune to the degradation caused by eddy currents [14]. These are particularly important merits for high-field cardiac applications where significant field inhomogeneities are usually present.

The imaging diagram for iMSDE based CVW imaging is shown in Fig. 1b. Benefiting from the short duration of the iMSDE sequence (∼20 ms), only 1 R-R interval was required for CVW imaging. The turbo field echo (TFE) acquisition window had a total duration of less than 120 ms. The trigger delay time ranged from 550–750 ms (Fig. 1b), based on subject heart rate. Other scanning parameters for the optimized iMSDE-prepared black-blood coronary vessel wall imaging protocol include: 3D TFE sequence, TR/TE 4.8/1.5 ms, flip angle 20°, TFE factor 19, FOV 300×264×30 mm, pixel size 0.9×0.9×3 mm, in-plane reconstruction matrix 512×512, fat saturation, and two averages. The total scan time was 4 min 6 sec with 100% navigator efficiency (Calculated for a heart rate of 60 beats/min).

### Feasibility Testing and Sequence Optimization

The feasibility of iMSDE CVW MR imaging was tested in 10 healthy volunteers (7 males, age range: 28–63 years) without known cardiovascular disease.

Localizing scans were obtained and the subject specific trigger delay and acquisition window were determined as previously described [15]. These scans were followed by coronary MRA using a bright blood three-dimensional (3D) TFE sequence in combination with spectrally selective fat saturation to image the right coronary artery (RCA) lumen. Imaging parameters were: TR/TE 6.2/3.1 ms, 15° flip angle , 10 slices; acquired resolution: 0.98×0.98×3 mm. The imaging slab was planned to cover the entire RCA up to the origin of the posterior descending branch. In the same orientation, the CVW scans of the RCA were acquired.

After feasibility was established, the first gradient moment of the iMSDE sequence was optimized to balance the blood suppression capability and overall image quality [13]. For this purpose a series of images with 1.5 ms dephasing gradient duration were acquired at the same location in 3 volunteers while changing the gradient strength, and correspondingly, the first gradient moment (m1). The m1 varied from 0–300 mTms^2^/m, in steps of approximately 75 mTms^2^/m. Cardiac synchronization was used to trigger the signal acquisition and respiratory navigator was used to compensate for involuntary motions caused by breathing. By using only 1 average, total scanning time was 2 min 3 sec without considering navigator efficiency.

Signal-to-noise ratio (SNR) was defined as the ratio between the signal of the region of interest (ROI) and the standard deviation of an ROI from myocardium [16]. Contrast-to-noise ratio (CNR) between two tissues was defined as the difference of the SNR between the tissues.

Due to the limited size of the coronary wall, the cardiac myocardium was selected for contrast-to-noise ratio optimization. The CNR between the left ventricle myocardium and blood, as well as the SNR of myocardium and blood were used for optimization.

### Reproducibility study

The feasibility of robust CVW MR imaging using the iMSDE sequence was tested in a reproducibility study in 19 healthy volunteers without any known symptoms of coronary atherosclerosis. In order to image a range of CVW thicknesses, 10 younger volunteers (8 males; mean age 25±4.7 years (mean ± standard deviation), as well as 9 older volunteers (7 males; mean age 59±9.2 years) were recruited.

A stack of images, parallel to each other, were planned based on the scout right coronary artery angiogram scan using three-point planning. The slice package contained 20 slices so that the entire target artery was covered, even when the arteries were tortuous.

To assess the reproducibility of the CVW MR imaging protocol, all subjects were imaged twice. After the first scanning session was complete, patients were removed from the magnet and the scanner table. A second session was initiated within 15 minutes after completion of the first. In the second scanning session, the entire exam was repeated, including patient and tabletop re-positioning, acquisition of scout views as well as a fresh determination of subject specific trigger delays followed by acquisition of the coronary artery luminogram and vessel wall images.

### Image processing and analysis

For viewing purposes, all coronary artery lumen and vessel wall images were processed with a curved multiplanar reformation algorithm by using the Soapbubble research tool (Soapbubble, Philips Healthcare) [17]. The curved multiplanar reformation process allowed for the simultaneous display of multiple coronary segments in one two-dimensional (2D) representation, to facilitate further image analysis. Two experienced MR reviewers with 7–10 years of cardiovascular image review experience (TL and SCG) then evaluated the reformatted images. The MR reviewers first evaluated the image quality according to the readability of the coronary arteriograms and vessel wall images on a three-point scale. A grade of 1 was given when images were uninterpretable due to motion artifacts and/or suboptimal suppression of luminal signal; a grade of 2 denoted acceptable image quality with only minor artifacts; and a grade of 3 was assigned in cases with good artifact-free image quality. For images graded with scores of 2 or 3, the black blood images were then evaluated for quantitative measurements.

For quantitative analyses, the RCA was divided into proximal and middle parts in accordance with AHA classifications [18]. Lumen and outer wall (if applicable) boundaries were manually delineated (reviewer: CJ, peer reviewed by SCG or TL) on the MR images using custom-written software (VesselMASS, Laboratory for Image Processing (LKEB), Leiden, The Netherlands). Lumen diameter and mean wall thickness of the vessel wall were measured based on the manual boundary delineations by VesselMASS: First, mean lumen diameter was measured in both proximal and middle segments, from both the bright and black blood images. Subsequently, visible parts of the vessel wall were outlined and mean anterior and posterior wall thickness was measured.

To evaluate the blood nulling consistency of the iMSDE sequence, the SNR of the left ventricle and the myocardium from both scans for all subjects (N = 16) were also measured, using the approach described above.

### Statistical Analysis

Mean image quality was compared using Wilcoxon signed rank test. The reproducibility of iMSDE CVW imaging was evaluated using Bland-Altman plots to investigate the segment-by-segment agreement between the two measurements. The agreement between two measurements with regard to mean lumen diameter and vessel wall thickness between different scanning sessions was compared using paired Student's t-test. Student's t-test was also used to compare the vessel wall thickness measurements between the younger and older groups. Wilcoxon signed rank test was also used to compare the ventricle and myocardium SNR between two scans. A P value below 0.05 was taken to indicate statistical significance.

## Results

### Sequence optimization

**Figure 2:**
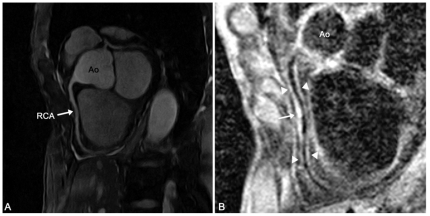
Results after sequence optimization in a 78 y/o male volunteer. A) Bright blood coronary MRA of the right coronary artery (RCA). The RCA can be visualized with excellent image quality. B) Good delineation of the vessel wall (arrowheads) with excellent suppression of luminal blood was obtained. Note the focal area of wall thickening (arrow).

**Figure 3:**
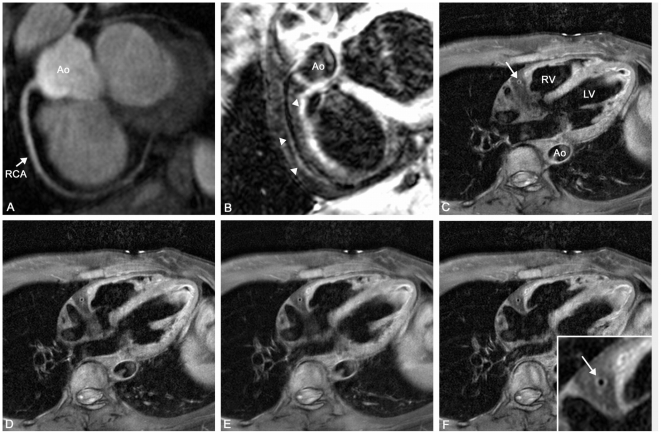
Results after sequence optimization in a 33 y/o female volunteer. A) Again, the right coronary artery (RCA) can be visualized with good image quality on bright blood coronary MRA. Ao indicates aorta. B) Good delineation of the vessel wall (arrowheads) with good suppression of luminal blood was obtained. C–F) demonstrate 4 cross-sectional iMSDE images in different areas of the RCA with good delineation of the vessel wall (arrow). RV indicates right ventricle, LV indicates left ventricle.

**Figure 4:**
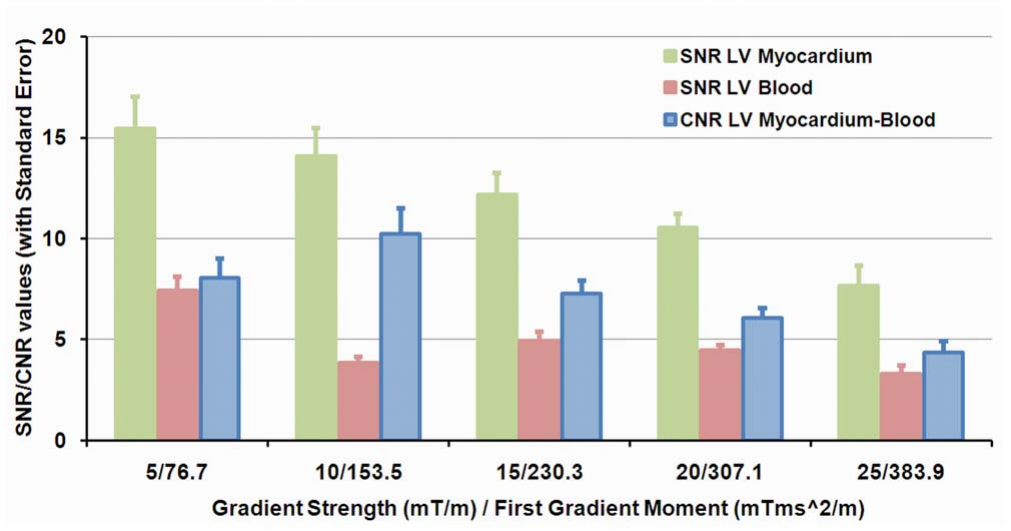
iMSDE sequence parameter optimization. SNRs of LV myocardium and blood and CNRs between blood and myocardium at different m1 values were shown. The CNR (Blue Bar) maximizes when the first gradient moment is 153.5 mTms^2^/m. The error bars indicates the standard error of all measurements.

iMSDE coronary vessel wall imaging was found to provide good image quality (Figs. 2 and 3). Lumen SNR demonstrated decrease with an initial increase of the dephasing gradient strengths and at the same time, the CNR between blood and myocardium was maximized when the first gradient moment was 153.5 mTms^2^/m as seen in Fig. 4. Further increases in gradient strength reveal loss of the myocardium signal without significant improvements in blood suppression. As a result, the optimal value of the first gradient moment based on CNR consideration (153.5 mTms^2^/m) was used in the following experiments.

### Reproducibility study

Successful measurements of the coronary lumen were obtained in 17 of 19 subjects. The other 2 subjects were excluded due to persistent problems with erratic vectorcardiograms.

For coronary vessel wall measurements, 12 (in 3 subjects) out of 136 potential vessel wall measurements (17 subjects×4 measurements at 2 time points) could not be obtained, due to VCG failure (n = 4), susceptibility artifacts (n = 4) or motion artifacts (n = 4), in 1 of the 2 acquisitions. Among the remaining 124 segments, 84 segments were identified and measured, 68 segments (34 pairs) were matched for the Bland-Altman analysis, and the 16 unmatched segments were not included in the Bland-Altman plots. The remaining 40 vessel segments exhibited no discernable vessel wall.

Image quality of the images was generally rated as ‘good and artifact free’ (grade 3 as defined above). Mean image quality of the proximal and middle segments of the RCA with both bright and black blood acquisitions is listed in Table 1.

**Table 1:** Image quality of the visualized proximal and middle segments in both the bright blood scan and iMSDE vessel wall sequence for both scanning sessions.

Sequence	Location*	Measurement 1 (mean ± SD)	Measurement 2 (mean ± SD)	*P*-value †
Bright Blood	Proximal (n = 17)	2.88±0.33	2.82±0.39	0.32
	Middle (n = 17)	2.94±0.24	2.94±0.24	1.00
Black Blood (iMSDE)	Proximal (n = 15)	2.63±0.50	2.44±0.51	0.08
	Middle (n = 15)	2.69±0.48	2.69±0.60	1.00

*Values represent number of paired measurements.

† Calculated using Wilcoxon signed rank test.

**Figure 5:**
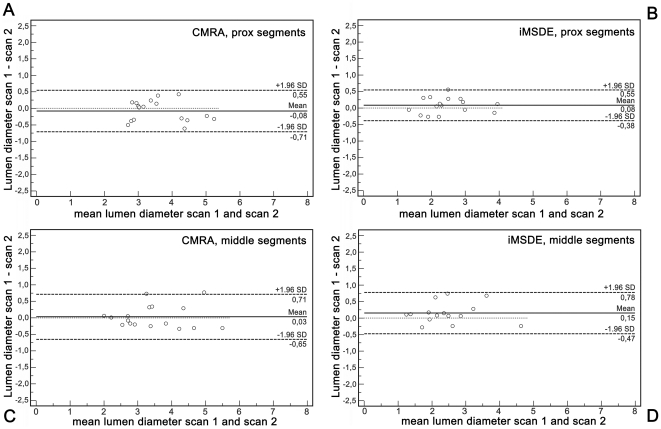
Agreement of lumen diameter measurements. Bland Altman plots for right coronary artery (RCA) lumen diameter as measured on the bright blood coronary MRA (A, C) and on iMSDE vessel wall scans (B, D) demonstrate low bias between results of the first and second scanning session. Measurements were performed in proximal (A, B) and middle (C, D) RCA segments.

**Figure 6:**
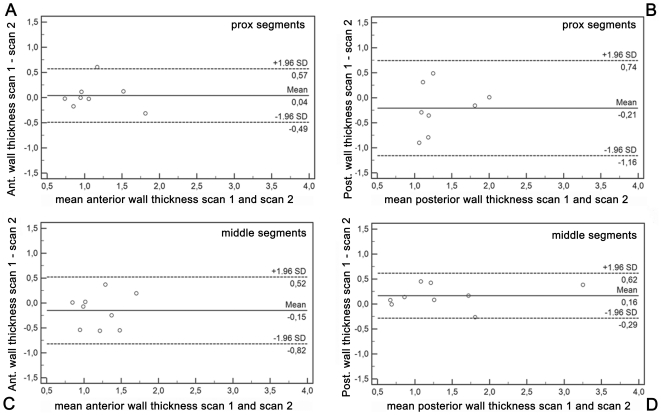
Agreement of vessel wall thickness measurements. Bland Altman plots for right coronary artery (RCA) vessel wall thickness measured on iMSDE images in both the anterior (A, C) and posterior vessel wall (B, D) demonstrate good agreement between results of the first and second scanning session. Measurements were performed in proximal (A, B) and middle (C, D) RCA segments.

Reproducibility of both the lumen and vessel wall measurements was good, as evidenced by similar mean luminal diameter and wall thickness at the two different time points (Tables 2 and 3), as well as small bias in the Bland-Altman analyses (Figs. 5 and 6). Of note, there was a substantial difference in luminal diameter between bright and black blood imaging (Table 2), most likely due to partial volume effects and human eye's natural sensitivity to bright objects.

**Table 2:** Reproducibility of lumen diameter measurements.

Sequence	Location*	Measurement 1 (mm ± SD)	Measurement 2 (mm ± SD)	*P*-value †
Bright Blood	Proximal (n = 17)	3.57±0.80	3.65±0.87	0.74
	Middle (n = 17)	3.47±1.02	3.44±1.00	0.14
Black Blood (iMSDE)	Proximal (n = 16)	2.49±0.75	2.40±0.73	0.23
	Middle (n = 15)	2.54±0.87	2.37±0.89	0.31

Measurements of lumen diameter (in mm ± SD) were performed on both bright blood coronary MRA and the black blood vessel wall scans of the first and second scanning session. There are no significant differences in absolute lumen diameter measurements.

*Values represent number of paired measurements.

† Calculated using paired Student's t-test.

**Table 3:** Reproducibility of Vessel Wall Imaging using the iMSDE prepulse.

Location*	Measurement 1 (mm ± SD)	Measurement 2 (mm ± SD)	*P*-value †
Anterior Proximal (n = 8)	1.15±0.37	1.16±0.43	0.92
Posterior Proximal (n = 8)	1.23±0.50	1.43±0.37	0.27
Anterior Middle (n = 9)	1.12±0.30	1.28±0.31	0.23
Posterior Middle (n = 9)	1.47±0.30	1.31±0.13	0.64

Measurements of vessel wall thickness (in mm ± SD) were performed in both the anterior and posterior vessel wall in proximal and middle right coronary artery segments. There are no significant differences in measured vessel wall thickness between the first and second scanning session.

*Values represent number of paired measurements. Only segments with discernable vessel wall were taken into account.

† Calculated using Student's t-test.

**Figure 7:**
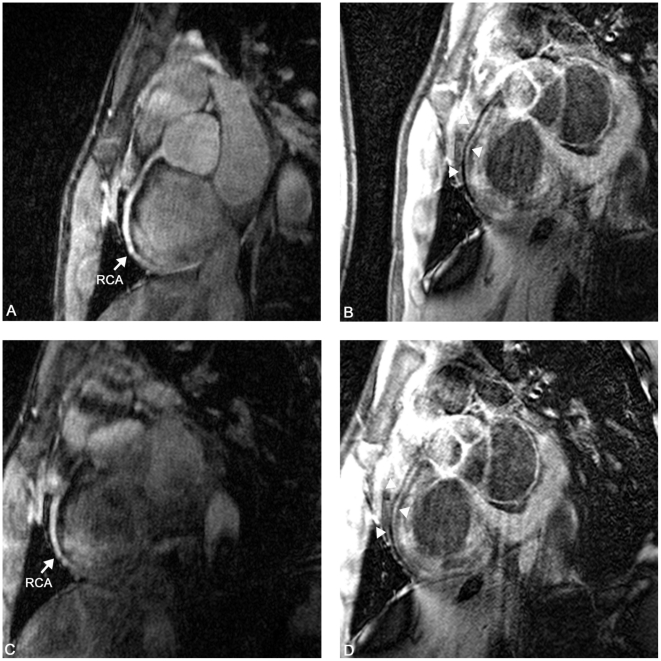
Bright blood coronary MRA and iMSDE vessel wall scan in a 23 y/o male. A and B represent the first scanning session, C and D represent the second session. The right coronary artery (RCA) was visualized in both sessions, and on the iMSDE scan there is good delineation of the vessel wall (arrowheads). Note excellent suppression of blood signal.

**Figure 8:**
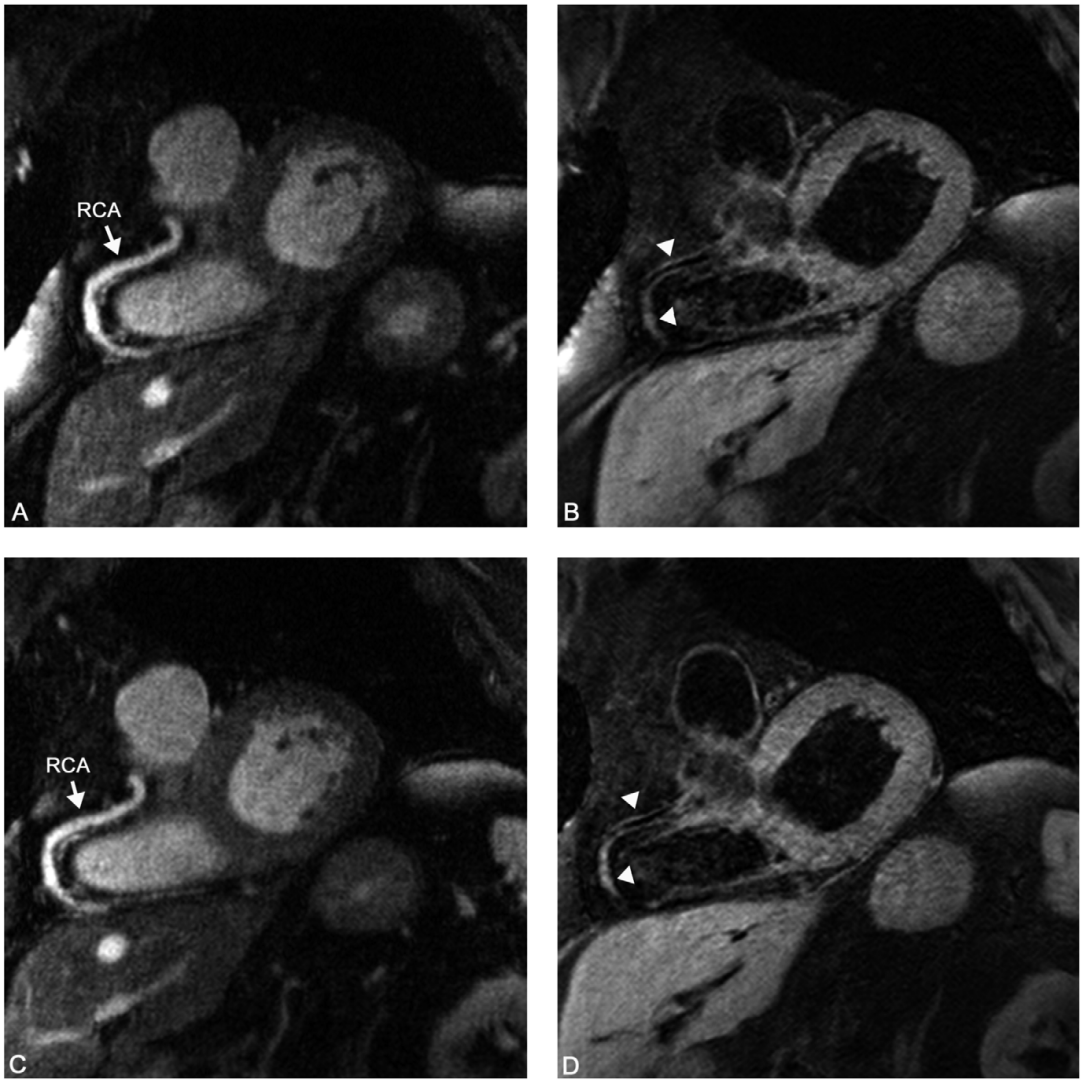
Bright blood coronary MRA and iMSDE vessel wall scan in a 76 y/o male. A and B represent the first scanning session, C and D represent the second session. Excellent visualization of the right coronary artery (RCA) (A, C) and vessel wall (arrowheads) (B, D) was achieved in both sessions. Note excellent suppression of blood signal.

Comparing the vessel wall thickness between younger and older subjects, a significantly thickened vessel wall was observed on in the older population (Younger: 1.09±0.34, Older: 1.69±0.37, p<0.01). Examples of CVW images at different time points in the younger and older age groups are shown in Figs. 7 and 8.

Comparing the blood suppression consistency of the iMSDE sequence, no significant differences were found between the two scans for both the left ventricle SNR (Scan 1: 3.86±1.19, Scan 2: 3.79±1.20, p = 0.5619) and myocardium SNR (Scan 1: 15.26±6.58, Scan 2: 16.83±6.25, p = 0.2312).

## Discussion

In this work we have demonstrated the feasibility and reproducibility of a novel and more time-efficient method of coronary vessel wall (CVW) imaging using the improved motion-sensitized driven equilibrium (iMSDE) technique.

Compared to the traditional DIR technique [11], the iMSDE technique is advantageous in a few respects. First, it has improved time efficiency due to the much shorter preparation time (iMSDE ∼20 ms vs. DIR 400–500 ms [13]). Since the iMSDE sequence does not rely on a long TI for blood suppression, it is more suited to coronary artery imaging applications. As demonstrated in this study, the iMSDE prepulse fits within a single heart beat, which allowed imaging with doubled time efficiency (i.e. half the acquisition time) when compared to DIR-based sequences. Second, iMSDE can suppress blood flow in virtually any direction and thus promises improved flow suppression efficiency, especially for tortuous arteries. The longitudinal images obtained in this study can delineate an extended coverage of the coronary artery clearly in a stack of thin slices, which will be very helpful for the early and fast diagnosis of any possible atherosclerotic plaques.

The reproducibility of the new technique has been shown to be good, as evidenced by the small bias and variation in the Bland Altman analyses. Furthermore, to the best of our knowledge, although a number of coronary wall thickness reproducibility studies have been reported on lower field-strength systems[19], [20], [21], this is the first report that has investigated reproducibility of CVW imaging at 3.0T. Good reproducibility is a key feature of any technique that is to be applied to follow changes in coronary wall geometry over time.

In iMSDE based MR scans, the orientation of the blood flow affects blood suppression efficiency and an individualized flow direction optimization would maximize the overall image quality. In this study, however, the inherently long acquisition time for coronary artery imaging prevented us from conducting a case-by-case optimization. Additionally, since the image acquisition plane was always planned in parallel with (using the 3-point planning available on the scanner) the RCA, the blood flow orientation caused suppression efficiency variation has been minimized. No special orientation measures have been taken to optimize the gradient strength in different directions. Ideally, the gradients along the artery dimension should be different from other directions for optimal performance. This approach, however, can be impractical for coronary artery imaging, since the artery itself is always tortuous and no individual direction could be defined as the main flow direction.

The current study has several limitations. In the reproducibility part of the study, only the RCA from a relatively small group of subjects were imaged due to the scanning time limitations. This fact, however, does not negatively affect the conclusion of this study since the primary goal of this study was to test the feasibility and reproducibility of the new iMSDE based CVW imaging technique. No technical obstacles are anticipated for transferring this technique to a larger population or for visualizing other coronary arteries. Another limitation of this study is that the study population has been limited to volunteers with no known coronary diseases. However, we anticipate seeing this technique more robustly applied to patients with diagnosed diseases, since the thickened coronary artery vessel wall generally allows for better visualization, particularly for a resolution and signal-to-noise limited application like this.

Also, the spatial resolution used in this study is relatively low, which prevented finer segments to be quantified using MRI CVW imaging. However, the primary goal of this study was to explore the feasibility and reproducibility of CVW imaging at 3T rather than optimizing the technique for all segments. The low resolution occasionally makes it difficult to visualize normal coronary artery walls due to partial volume effects. A higher SNR generally allows higher spatial resolution images to be used and the vessel wall is more robustly imaged, as has been demonstrated by Gerretsen et al who have described acquired voxel sizes of 0.78×0.78×2.0 mm^3^ [22]. Advances in hardware such as a 32-channel cardiac coil and the recently proposed parallel transmission technique [23] have both been demonstrated for higher SNR imaging. We anticipate that these technical advancements will further improve the current coronary artery imaging technique by increasing the current spatial resolution used.

Finally, this study lacks a direct comparison between iMSDE and DIR based CVW imaging due to the lack of a proper DIR implementation at our institution at 3.0T. The shorter magnetization recovery period (a single R-R) used by iMSDE allows for much improved acquisition efficiency as well as more flexible trigger delay selection, compared to the multiple R-R intervals used by the DIR technique. This approach, however, will also lead to reduced magnetization recovery during each heartbeat, thus limiting the overall SNR. Theoretically speaking, by averaging two acquisitions of iMSDE acquired with shorter TR, the overall SNR should still be higher than the single acquisition of DIR with longer TR. The improved flow suppression [13] and lack of T1 dependency also justify the usage of this technique.

Although the iMSDE technique has been shown to provide robust coronary vessel wall delineation in this study, the clinical application of coronary vessel wall imaging still faces challenges. Except for the high spatial resolution requirement discussed above, to achieve robust and reliable respiratory motion tracking also represents challenges for certain subjects. A number of new techniques have been recently proposed to improve the robustness and efficiency of respiratory motion tracking and they are also expected to help coronary vessel wall imaging applications [24]. Besides, to achieve robust cardiac gating at the presence of arrhythmia can also be difficult. In this study, an arrhythmia rejection algorithm [15] was adopted to automatically reject data acquired during arrhythmia and has been found to help improve image quality.

In conclusion, we have demonstrated the feasibility and reproducibility of a new method to improve the time-efficiency of coronary artery vessel wall imaging. The reproducibility of the study has also been well established in a group of volunteers in two age groups. This study paves the way to further test this technique in a clinical environment for fast and accurate coronary artery disease detection.
